# How do nurses support chronically ill clients’ participation and self-management in primary care? A cross-country qualitative study

**DOI:** 10.1186/s12875-022-01687-x

**Published:** 2022-04-18

**Authors:** Kerstin Hämel, Gundula Röhnsch, Marcus Heumann, Dirce Stein Backes, Beatriz Rosana Gonçalves de Oliveira Toso, Ligia Giovanella

**Affiliations:** 1 Department of Health Services Research and Nursing Science, School of Public Health, Bielefeld University, Universitätsstraße 25, 33615 Bielefeld, Germany; 2 Qualitative Social and Education Research, Department of Education and Psychology, Free University of Berlin, Habelschwerdter Allee 45, Berlin, 14195 Germany; 3 Franciscan University – UFN, Rua dos Andradas, 1614, Centro, Santa Maria, RS CEP: 97010-030 Brazil; 4 Center of Biological and Health Sciences, Western Paraná State University - UNIOESTE, Rua Universitária, 1619, Jardim Universitário, Cascavel, PR CEP 85819-110 Brazil; 5 National School of Public Health, Fundação Oswaldo Cruz, Av Brasil 4036 s. 1001, Rio de Janeiro, RJ CEP 21040-361 Brazil

**Keywords:** Chronic diseases, Self-management support, Primary care nursing, Empowerment, Primary health care, Long-term care, Person-centered care, Germany, Brazil, Spain

## Abstract

**Background:**

In the context of the advancement of person-centered care models, the promotion of the participation of patients with chronic illness and complex care needs in the management of their care (self-management) is increasingly seen as a responsibility of primary care nurses. It is emphasized that nurses should consider the psychosocial dimensions of chronic illness and the client’s lifeworld. Little is known about how nurses shape this task in practice.

**Methods:**

The aim of this analysis is to examine how primary care nurses understand and shape the participation of patients with chronic illness and complex care needs regarding the promotion of self-management. Guided interviews were conducted with nurses practicing in primary care and key informants in Germany, Spain, and Brazil with a subsequent cross-case evaluation. Interpretive and practice patterns were identified based on Grounded Theory.

**Results:**

Two interpretive and practice patterns were identified: (1) Giving clients orientation in dealing with chronic diseases and (2) supporting the integration of illness in clients’ everyday lives. Nurses in the first pattern consider it their most important task to provide guidance toward health-promoting behavior and disease-related decision-making by giving patients comprehensive information. Interview partners emphasize client autonomy, but rarely consider the limitations chronic disease imposes on patients’ everyday lives. Alternatively, nurses in the second pattern regard clients as cooperation partners. They seek to familiarize themselves with their clients’ social environments and habits to give recommendations for dealing with the disease that are as close to the client’s lifeworld as possible. Nurses’ recommendations seek to enable patients and their families to lead a largely ‘normal life’ despite chronic illness. While interview partners in Brazil or Spain point predominantly to clients’ socio-economic disadvantages as a challenge to promoting client participation in primary health care, interview partners in Germany maintain that clients’ high disease burden represents the chief barrier to self-management.

**Conclusions:**

Nurses in practice should be sensitive to client’s lifeworlds, as well as to challenges that arise as they attempt to strengthen clients’ participation in care and self-management. Regular communication between clients, nurses, and further professionals should constitute a fundamental feature of person-centered primary care models.

## Background

The task of guaranteeing person-centered care for an increasing number of people with chronic illness has become a core challenge for healthcare systems all over the world. Yet, by its very nature, chronic illness poses complex challenges for self-management to these patients. For many of them, life is a constant process of balancing disease-specific requirements and everyday tasks in school, at work, and in their families [1, 2]. A vital task of primary health care professionals, then, is to support people with chronic illness in leading as normal a life as possible [3]. One aspect of this support is controlling disease symptoms as far as possible and strengthening health resources to prevent or delay the progress of the disease [4, 5]. This also requires people with chronic conditions to actively participate in their care by considering therapeutic recommendations or adapting their lifestyles accordingly. However, due to extensive functional impairments or psychosocial challenges resulting from their illness, these patients and their families can sometimes be overwhelmed by such a proactive role in care [6, 7].

For the chronically ill, primary care is the first and continuous point of contact in the healthcare system [8]. Particularly in more recent care models, these are the nurses who often encourage patients with chronic illness to participate in their own care by enhancing their health literacy [9, 10] and promoting self-management [11–13]. Nurses thus assume the role of personal point of contact in care-related matters.

Within primary care teams, nurses frequently maintain especially close contact to chronically ill patients and their relatives. As a result, they see themselves in a strong position to promote patient self-management [13]. Nurses regard clients’ participation in their care as a precondition for more health-conscious behavior and greater willingness to assume more responsibility for their own health; client participation is also seen as a basis for increased patient satisfaction [14]. Furthermore, study results indicate that where nurses are able to define the scope of their own tasks in the provision of primary care, they make a significant contribution to promoting client participation [15]. When nurses approach the task of promoting patient self-management by taking into account the psychological and social impacts of chronic illness on their patients, this can enable clients to lead a higher quality of life despite their disease. To this end, nurses facilitate their patients’ personal development and ability to define their own values and priorities in life, which go beyond their continual efforts at ‘disease control’ [16, 17]. Nurses' orientation towards clients' life circumstances is one of the core aspects in this regard. At the same time, nurses must accept that individual clients may not want to ‘optimize’ their self-management and health literacy, in effect, that they are not interested in participation [14].

Theoretical-conceptual approaches of nursing science, too, increasingly emphasize the lifeworld dimension of nursing [18]. In their concept of a “lifeworld-led care” [19, cf. 20] Todres, Dahlberg and Galvin focus on the individual experiences of care recipients [19]. According to this approach, patients and nurses are to design care as partners finding ways to acknowledge each other’s competencies [20].

To date, the strategies used by primary care nurses to promote self-management and the participation of those with chronic disease and long-term care needs has been examined predominantly within the framework of intervention studies. Less attention has been paid to how this can be implemented in routine care [21]. The few existing studies on this subject also reveal a series of inhibiting factors: Nurses are unsure, for example, to what extent they can or should accept patients’ decisions that could adversely impact their health [12, 22].

At times, nurses have a rather limited understanding of client participation, restricting it to the clients ‘complying’ with nursing recommendations and adopting a health-promoting lifestyle [23, 24]. Qualitative studies also show that nurses sometimes attribute complications in the patient’s condition to their lack of motivation to acquire knowledge of their disease [13, 23]. Nurses who are oriented towards a bio-medical model of disease often tend to unilaterally emphasize disease management, such as the adequate use of medication, while ignoring psycho-social support in dealing with emotional, disease-related challenges [25, 26].

Studies also show that – depending on the design of primary health care and health policy in the respective countries – nurses in different countries are equipped in different ways for carrying out those tasks [27, 28]. In some countries, for example in the Anglo-Saxon countries, Scandinavia, as well as Spain [29] and Brazil [30, 31], primary health care nurses are increasingly required to support the participation of clients in their own care by strengthen their health literacy and self-management. In contrast to this, primary care in Germany [27] and many Eastern European countries [28] is in its early stages of involving nurses in this task. It is therefore important for the development of person-centered primary health care models to obtain knowledge of the strategies nurses employ to support chronically ill clients’ participation and self-management in primary care.

## Methods

### Aim of the study

The aim of this study is to investigate how primary health care nurses understand and shape the participation of patients with chronic illness regarding the promotion of self-management. The focus of this analysis lies on how nurses’ understanding of client participation affects the strategies implemented in carrying out this task. This particular focus serves to identify the conditions advantageous for fostering client participation in primary care as a nursing task. The results of the study form a basis for the design of person-centered primary care models.

### Study design

This analysis is part of a cross-country, qualitative interview study. It is based on expert interviews as a special form of guided interview [32]. We questioned nurses practicing in primary care, as well as key informants having expert knowledge of the practices of primary health care nurses. We consider care models in three different countries – Germany, Spain, and Brazil – to increase the validity of our findings on a broad empirical basis taking into account different health systems and primary health care models. To this end, we included experts from countries, whose primary health care nursing is at different levels of development. The interviews were evaluated from a cross-country perspective based on Grounded Theory [33] with the aim of highlighting interpretive and practice patterns [34].

### Research setting: primary care models in Germany, Spain, and Brazil

In *Germany,* primary care is the responsibility of general practitioners, who usually work in private, single or joint practices. Medical assistants are usually part of these practice teams [27]. Since 2004, it is legally permissible to establish “medical care centers” which allow for the integration of different medical subdisciplines within a single organization. These centers can include primary care physicians (GPs and pediatricians) as well as other specialists. They also allow for closer cooperation with non-medical primary health care providers, including home care nursing services or pharmacies [35, 36]. Multi-professional primary health care teams involving nurses are only rarely established in standard care but are being tested increasingly (partially as pilot projects) in some regions [3, 37]. There are projects, too, in which different health professions cooperate to provide primary care while practicing in institutionally distinct facilities (such as family doctor practices and home care services). The varying approaches investigated for this study reveal different task profiles of nurses. These include the delegation of doctors’ tasks to nurses, e.g. the assessment of diagnostic parameters, provision of home visits, or care coordination. In addition, the study includes models that comprise the establishment of additional services to improve the support and care of chronically ill patients in different phases of life, such as the promotion of self-management, health counseling, and psychosocial support.

In contrast to Germany, the national health service of *Spain* (SNS) has been involving nurses as an inherent part of multi-professional primary health care teams since the end of the 1980s [38]. Nurses practice in mainly public primary health care centers (Centros de Salud) [29, 39]. As a rule, general practitioners and family and community nurses normally form a tandem, providing care to a common patient base. Nurses assume responsibility for the regular monitoring of patients with chronic conditions and analyze the overall health situation, as well as health-related behavior, in regular follow-up visits [40]. On this basis, they develop self-management strategies together with clients and their families [29]. Predominantly, nurses perform individual patient consultation in the health centers but, if required, also visit patients and their families in their home environment. In addition, they offer group-based health promotion and disease prevention opportunities (such as diabetes groups, physical activity groups) [29, 30]. Advanced Practice Nursing is also increasingly available, where nurses as case managers, for example, ensure comprehensive care for complex, chronic conditions [41].

In Brazil, too, multi-professional teams practice in primary health care centers. Pursuant to the national Family Health Strategy (FHS), launched as a federal program in the 1990s, the family health teams investigated for this study are responsible for the primary care of the population in geographically defined areas. Each family health team comprises 1 physician, 1 nurse, 1–2 technical nurses, and 5–6 community health workers [42]. The method of operation of each team is oriented on the family and community [43]. Nurses are usually responsible for the team coordination and assume a series of organizational and management roles at the centers, accordingly. In addition, they perform duties in the field of health promotion and disease prevention. This is the area where the focus of nursing lies particularly on family and community, where nurses perform home visits, supervise self-help groups, and coordinate the work of community health workers and technical nurses. Group programs for the chronically ill and other vulnerable groups (e.g. older people with functional limitations) are usually headed and coordinated by nurses [43]. While the work of nurses has focused traditionally on mother and child health, the scope of duties for which they are responsible has recently extended to include the individual support and care of clients with chronic conditions − putting the work of consultation ever more at the forefront of their duties.

### Sampling and field approach

In order to account for the regional differences in the design of primary care within the three countries, as well as to compile as broad a representation of primary-care nursing practice as possible, we interviewed nurses and key informants from seven (7) German federal states, four (4) autonomous communities in Spain and four (4) Brazilian federal states. A preselection of the regions and facilities was made based upon our previous research and knowledge of regional differences as well as recommendations of cooperation partners (see acknowledgements). Some study participants were recruited with the support of nursing associations and other cooperation partners; others were considered on recommendation of our interview partners. The acquisition of sufficient (and varying) primary care facilities and pilot projects for the study was particularly challenging in Germany, since, as explained above, these are not prevalent there. Insofar, we performed a thorough review of pilot projects to date to define the scope of currently practiced approaches and were able to recruit members of these project teams as study participants. Our study sample comprises a total of 57 persons – 34 nurses and 23 key informants (Table 1). Key informants were nursing scientists, decision makers in the areas of nursing and primary health care on a regional and national level, as well as specialists assuming coordination tasks on behalf of primary care facilities. With few exceptions (*n* = 3 persons), key informants are also qualified nurses. We included only certified nurses,^1^ no nursing assistants or persons with other qualifications, as well as nurses with further professional or specialized training, and nurses with higher academic degrees (master’s and doctorate).

**Table 1:** Study sample

Group	Total	Country			Sex	
		Germany	Spain	Brazil	Male	Female
Key informants	23	6	7	10	5	18
Practicing nurses	34	12	12	10	3	31
Total	57	18	19	20	8	49

### Data collection

The interviews were conducted between August 2019 and December 2020 and lasted between 20 and 132 min. We held forty-four (44) one-person interviews (*n* = 1), five (5) two-person interviews (*n* = 2), and one (1) three-person interview (*n* = 3). Face-to-face interviews were held exclusively in Brazil (KH, DSB, LG, BRGOT). Due to the Covid-19 pandemic, interviews in Spain (MH) and Germany (GR) had to be conducted as a mixture of face-to-face interviews or interviews by telephone or Zoom. All study participants gave their written informed consent in advance. The interviews in Brazil and Spain were conducted predominantly by native speakers in Spanish or Portuguese. The interview team included two (2) of the study’s co-authors (BRGOT, LG), two (2) nursing students, two (2) PhD candidates, and one (1) postdoctoral researcher. Two (2) of the interviewers in Spain were not Spanish native speakers but had high levels of Spanish-language proficiency. All interviewers were trained by the core research team of this study (KH, GR, MH) and closely supervised during the interview phase. With the exception of two (2) cases, one member of the core research team was present during the interviews in order to ask questions of comprehension about the interviewees' answers or ask for further detail to aspects of the questions defined in the guideline. This was especially important for the clarification of specific terminology and its meanings in the context of the respective health care system and, in turn, to improve the comparability of the interviews between the different countries. It also enabled the core research team to continuously exchange information about interview processes and findings from interviews in the different countries and to ensure the quality of data collection.

The interviews sought to gather information about the nurses’ “context knowledge” of the living conditions of clients with chronic illness and complex care needs, as well as about their “process knowledge” regarding the design of nursing activities [32 p. 471]. The interview questions were designed according to the episodic interview approach [44, 45]. Targeted questions were combined with narrative questions that required respondents to describe specific situations in which they experienced certain phenomena. This approach made it easier for interviewers and interview partners to reach a common understanding of the study’s central concepts.

The guidelines for the interviews with practicing nurses and key informants comprised the following themes: a) tasks of nurses in primary care and interprofessional collaboration; b) promotion of participation of clients with chronic conditions; c) promotion of client participation in group programs and in the community; d) conditions that promote/inhibit the strengthening of client participation as one of the nurses’ tasks.

For the interviews in Spain and Brazil, the guidelines were translated into Spanish and Portuguese. All interviews were recorded and transcribed in their entirety in the original language and pseudonymized. The Spanish and Portuguese transcripts were then translated into English. The translation of all Spanish transcripts and some of the Portuguese transcripts was done by the postdoctoral researcher who was also part of the interview team. The other Portuguese transcripts were translated in a course for English translators at UNIOESTE and by one of the student interviewers who was a native speaker and had a very good command of English. All translations were then carefully reviewed vis-à-vis the original transcript by one of the co-authors.

### Data analysis

In a first step, we performed computer-assisted coding of all transcripts, using MAXQDA software, according to the case-specific subject matter [44]. For this purpose, the core research team (KH, GR, MH) developed key categories deductively based on the themes in the interview guidelines and allocated the data to these descriptive categories. Prior to our data analysis, we conducted a thorough review of existing concepts and theories on the ways nurses support chronically ill clients in their self-management and participation. Here, we were also able to draw on our own integrative review, conducted in the context of this study [46]. This thorough study of available literature was essential to our ability to make conceptually sound claims based on our own data and, insofar, to position our conclusions with theoretical sensitivity [47, 48].

As a next step, GR and MH openly coded the categories relevant to the questions by addressing generative, sensitizing questions in close coordination [33, cf. 44] to the text. This enabled the inductive identification of subcategories. The resulting category system was discussed regularly with KH until consensus was reached on any ambiguous points.^2^ The subcategories were used to develop cross-case comparative dimensions (e.g. extent of agreement of self-management recommendations with the client’s lifeworld). Based on these dimensions, we then identified the similarities and differences between the individual cases (= interview partners) [49 p. 91 et seqq.]. Finally, we grouped the cases along the dimensions and their characteristics and analyzed them comparatively according to the appearance of specific feature combinations. We initially examined the cases of one group by means of case contrasting as to their similarities and subsequently conducted case comparisons between the groups in order to identify the differences [49 p. 96 et seqq.]. In the end, this procedure resulted in the identification of two interpretive and practice patterns, which we analyzed and interpreted according to their contextual meaning and under consideration of conditions, contexts, interactions, or consequences of phenomena [cf. 33, 49 p. 105 et seqq.].

## Results

Two interpretive and practice patterns can be identified: *“giving clients orientation in dealing with chronic diseases”* and “*supporting the integration of illness in clients’ everyday lives*”. These patterns are distinguished by the extent to which nurses – from the distinct perspectives of our two different interview partners, i.e. the nurses' own perception of their actions, or the key informants’ perceptions of nurses actions – (are willing to) engage with clients and their relatives and to consider the client’s lifeworld as well as values, preferences and needs, when encouraging their participation and self-management in primary care.

### Giving clients orientation in dealing with chronic diseases

Interview partners^3^ allocated to this pattern believe that many chronically ill persons are comparably seldom knowledgeable about their illness. Experience has taught them that patients are unsure about the best strategies for dealing with disease-related challenges and burdens. Patients are apparently unfamiliar with available therapy options and, moreover, neither they nor their families are aware that they can play an active part in their care.

“*(…) many chronically ill people do not know (…) that they and also their relatives can get involved in the scope of their care (…) for example, a patient, client with a stroke, what type of therapies are available, what he can do himself*.” (Nurse, Germany, E10:35)

Interview partners allocated to this pattern believe that a nurse’s task is to provide enough detailed information to clients to empower them to make decisions regarding their own care.

“*It is essential that they have all the information to be able to decide.*” (Key informant, Spain, E8:75)

These interview partners, both nurses themselves and key informants, regard nurses as experts who, from their professional perspective, can show clients and their families how to deal ‘correctly’ with chronic illness. Nurses impart skills, e.g. in courses, that help to prevent disease-related complications. Interview partners indicate that such skills are a prerequisite for clients’ ability to make decisions about their care together with their health care providers: *“(…) through the health education the individual can be involved in his/her selfcare*” (Key informant, Brazil, E9:22). However, nurses are doubtful that the courses they offer patients also address the vulnerable groups who need them most. Patient training courses are designed to convey knowledge and, according to our interview partners, appeal mainly to those who already take a proactive role in dealing with their illness. As a rule, these patients are aware of their needs and willing to take initiative to visit courses offered at various institutions.

*“(…) the patients whom we visit at home, only very few of them attend the courses, well, they are patients, who, let’s say, are in the midst of their lives, who have a strong interest in getting to know their disease and be able to deal with it with as less hardships as possible.”* (Nurse, Germany, E12:38)

Interview partners believe that one of a nurse’s tasks is to inform patients of support measures and services (e.g. adaptation of their living environments). They emphasize, however, that it is up to clients to decide which recommendations they would like to implement. Insofar, interview partners regard nurses as neutral “*adviser[s]*” (Key informant, Spain, E2:81). Interview partners allocated to this pattern emphasize that they must accept it if clients decide against certain recommendations made by nurses or continue to do things that are likely to adversely affect their health. At times, however, nurses then feel that they ‘failed’ to adequately demonstrate the purpose of recommended aids or the usefulness of certain practices. Yet, they feel they have no right to ‘force’ recommended aids or practices on clients who do not really agree with them. For these interview partners, client participation in their own care means allowing clients to decide independently how best to deal with their chronic illness and to which forms of support they take recourse in dealing with their long-term care needs.

Interview partners allocated to this pattern allow patients wide autonomy in dealing with their chronic illness by ‘staying out of’ their decisions. They emphasize patients’ personal responsibility, without giving serious consideration to the additional strain assuming such responsibility may cause patients. In their eyes, nurses to not bear responsibility for any potentially negative consequences patients may bear as a result of their own decisions. Nurses, thus, also protect themselves from emotional stress caused by an eventual negative course of the patients’ disease and their care. At the same time, they reassure themselves that at least they had informed patients of the consequences of not following their recommendations.

“*(…) it also happens that he does not want to accept any offers for assistance, although he’s at the end of his tether (…) at some stage I have to tell myself ‘Okay, he’s aware of it’, and I cannot change it*.” (Nurse, Germany, E8:49)

Interview partners allocated to this pattern rarely address the impact of chronic illness on the everyday lives of clients and their families, nor do they consider the extent to which the regular practice of certain therapy recommendations poses a challenge to them. Moreover, none of the nurses interviewed discussed aligning their recommendations with patients’ cognitive, social and/or financial resources, nor did they talk about tailoring their communications to ensure that patients were able to ‘understand’ their message.

### Supporting the integration of illness in clients’ everyday lives

Interview partners allocated to this pattern prefer to regard patients as actors and cooperation partners in primary care. They emphasize that nurses should take clients seriously in their subjective perception of their condition and their practice strategies. They assume that the way clients and their families deal with their health limitations is subjectively reasonable. This also includes decisions about if and how they implement recommendations of nurses in everyday life or if they avoid facing their illness.

From the perspective of these interview partners, it is important that nurses adjust their support to the individual needs and preferences of their patients. They emphasize that clients often know best what is good for them. In order to support their clients, it is essential that nurses actively listen and step back from their own stock of professional experience and knowledge.

“*(…) we have the tendency to think that since we are professionals, we know what must be done and the patient does not. Of course, many times, we have to be silent, and we have to listen much more than talk*.” (Nurse, Spain, E4:87)

These interview partners believe that the goal of nursing support should be to make everyday life with a chronic illness easier for patients and their families, so that they can lead as normal a life as possible. From the perspective of these interview partners, holistic care, support, and advice is therefore the only viable approach. Holistic care comprises more than the mere control and management of clinical symptoms. Here, nurses must consider the social circumstances and environments of their patients’ lives. Insofar, it is also important that nurses are networked in the communities where they practice in order to implement such a comprehensive assistance approach that addresses clients’ unique difficulties and needs. Nurses work together with providers of medical-nursing services (general practitioners and specialist physicians, therapeutic professionals, home care services) as well as with providers of social care (including volunteers and self-help organizations):

*“(…) that the patients, that they have everything they need (…) that is seeing the whole picture (…) when they are lonely that you put them in touch with volunteers, that there is somebody to take a walk with, to read aloud*.” (Nurse, Germany, E6:83)

On the basis of mutual trust, nurses endeavor to give patients an understanding of a health-promoting approach to their condition. To this end, they familiarize themselves with the patient’s habits and lifeworld, e.g. through home visits. These interview partners believe (mutual) trust encourages clients to ‘open up’ and talk about their experiences with their illness. As a result, nurses are able to make individualized recommendations suited to the circumstances of the patient’s daily life. In addition, they can better explain the goal of therapeutic measures. Nurses try to negotiate with clients about what type of health-relevant behavior is appropriate and also subjectively acceptable to the client. At the same time, they also try to ritualize commitments and ‘determine’ target agreements.

“*(…) the user is the main actor of the care plan. He will make a deal, with the specialist, signing a care agreement about his commitment of what he can do. At this moment it is time to discuss about what he can do, and what he will commit to (…).*” (Key informant, Brazil, E12:68)

For these interview partners, it is essential that nurses ‘get the users on board from where they are’. Insofar, they see it as self-evident that nurses therapy recommendations must be adapted to the client’s lifeworld and circumstances so that they can be realistically implemented. This also means putting recommendations in perspective and focusing increasingly on “*damage mitigation*” (Nurse, Brazil, E16:26) in dealing with chronic illness. These interview partners find this acceptable if it reduces the patients’ health-related risk.

However, nurses wishing to promote more extensive client participation in primary care are often confronted with ethical challenges. They frequently perceive it as a balancing act to consider what sort of lifestyles they can accept and how much autonomy of ‘their’ patients they can answer for. Patients’ refusal of offered support is often accompanied by nurses’ emotional dismay and professional helplessness. This is particularly true when patients are in the terminal phases of chronic illness, as one key informant remembers from his/her time as a practicing nurse.

”*I remember a patient with pancreatic hernia. He was cachectic at home (…) it was a sense of helplessness: He was being dehydrated, refused to eat (...) he said to me “[Antonia], you stayed calm; I want to die like that (…).”* (Key informant, Spain E5:65)

These nurses believe that they cannot force patients to adopt particular health behaviors, that they are not entitled to dictate to patients how to live, especially as this often has far-reaching implications, “*(…) I cannot decide the life of this person.*” (Nurse, Brazil, E5:145). From this perspective, it is inappropriate to give directive recommendations. This warrants emphasis, as there is rarely only ‘one’ correct decision in favor of or against therapeutic measures – when faced with an advanced stage of chronic illness, there may, in fact, be none at all.

“*There is this young man, for example, who suffered from bowel cancer (…) it was detected quite late, so no great chances (…) and after the last chemotherapy, for example, he said he was not going back [there]. We accepted this initially (…) that we always tell the patient 'Whatever you decide, we are at your side. We do it the way you want it' (…).*” (Nurse, Germany, E9:89)

Interview partners of the different countries involved in this study can be distinguished from one another by the area in which they believe nurses should strengthen client participation. For interview partners in Brazil and Spain, health-promoting practices, such as food choices and physical exercise, are seen as suitable measures to positively influence chronic illness. However, they are aware that many clients have difficulty implementing such practices given their sometimes rather precarious life circumstances, including the lack of financial means to buy certain foods and lack of opportunity to do sports. These nurses, thus, look for alternatives to the ‘best’ health-promoting behavior patterns.

“*(…) we know the oilseeds that we want the patient to consume are expensive, so in this sense what I got was that she consumed avocado more often, a little cheaper olive oil, and flaxseed, flaxseed will also help raise the HBL and it is cheaper*.” (Nurse, Brazil E8:16)

By contrast, interview partners in Germany find that precarious life circumstances are less likely to affect adequate treatment of chronic illness. These partners, instead, point to clients’ unwillingness to change ingrained and well-loved routines in order to implement new health care recommendations. This is the case, for example, when side effects of medication prevent them from maintaining accustomed day-to-day routines. Nurses in Germany find it a particular challenge to assess the extent to which they should or could adapt their recommendations to clients in order to enable their social participation despite a chronic illness.

## Discussion

The results of our study reveal two characteristic interpretive and practice patterns of how nurses promote the participation and self-management of chronically ill patients in primary care: nurses to whom the pattern ‘*giving clients orientation in dealing with chronic diseases*’ applies put emphasis on client autonomy. These nurses rarely intervene when clients adopt ‘inadequate’ strategies for dealing with their condition. Nurses rationalize their lack of intervention by arguing that they have at least made clients aware of the consequences of such behavior. Nurses to whom the pattern of ‘*supporting the integration of illness in clients’ everyday lives’* applies also attempt to give clients as much decision-making autonomy as possible. At the same time, however, they find it important to negotiate a viable strategy for dealing with chronic illness together with their clients. Therapeutic recommendations are adapted to patients’ life circumstances, in particular, allowing them to integrate their illness into their everyday lives, as has been consistently described in pertinent studies [50, 51].

A comparison of these patterns to typologies of other studies is revealing: One study by van Hooft and colleagues assigned nurses from different settings to four perspectives on the promotion of self-management among chronically ill patients [52]. One perspective described nurses as ‘coaches’: Similar to our pattern ‘giving orientation’ of our study, this perspective emphasizes client responsibility and decision-making power in dealing with their illness. Comparable to the pattern ‘supporting integration,’ it is important for nurses acting as ‘coaches’ to withhold their own professional views. They adjust the support they provide to the clients’ needs. Here, clients are regarded as experts on their illness. Unlike the study by van Hooft, et al., however, our own revealed that remarkably few nurses held paternalistic attitudes, which presume the superiority of professional over ‘amateurish’ knowledge [52]. Nor did the (self-)descriptions of our interview partners reflect an economistic view, according to which self-management is an especially important factor of cost minimization in the health sector [52].

Our results show that nurses seeking to promote the participation and self-management of clients with chronic illness and complex care needs, often face the challenge of balancing the clients’ decision-making autonomy with, in their view, optimal medical and nursing outcomes. Such conflicts have been consistently described in other studies [12, 22, 53]. Analogously, our study also reveals that nurses tend to assess the value of medical-nursing outcomes, resulting, for example, from health-promoting behaviors, higher than the clients’ freedom to decide in favor of or against care providers’ recommendations. When nurses do accept patient behaviors that may be, in their view, damaging to their health or perhaps even dangerous, it is only because they do not see any other options for intervention.

Especially our interview partners allocated to the pattern ‘supporting integration’ find it important to provide clients emotional support, as well. As clients’ illness progresses and becomes increasingly severe, nurses often suffer vicariously with them. In this regard, Delmar describes the risk that too close an emotional bond to patients’ ‘fate’ can pose, leading to nurses’ tendency to overprotect clients [54]. This contributes, in turn, to paternalism in nursing care. The nurses interviewed in our study respond to this dilemma by involving clients in their care and negotiating care strategies based on profound knowledge of their patient’s lifeworld and mutual trust.

Our study also indicates that the systemic-structural level of health care has an influence on how nurses understand and design client participation in primary care.

1. Predominantly interview partners in Brazil emphasized the aspects of health promotion and disease prevention as part of their efforts to promote client participation and self-management. Our results indicate that this can be explained by the role nurses assume in primary care within the Brazilian Family Health Strategy, wherein they bear responsibility for the implementation and organization of health promotion and prevention. They socialize closely in the lifeworlds and communities of their clients [43]. Other studies conducted in Brazil have also revealed that bonding with patients forms part of a professional strategy of nurses in family health teams [55, 56].
2. Our interview partners in Germany emphasize the duty of nurses to provide individualized support for clients dealing with chronic illness. Here, nurses guide clients in dealing with the challenges of their illness. These interview partners, however, refer relatively seldom to the task of health promotion and prevention in the context of the client’s lifeworld. This correspondents to the findings of nursing scientists in Germany, who criticize that disease prevention and health promotion are only gradually being incorporated in the task profiles of nurses in Germany [3, 57–59].
3. Our interview partners in Spain also emphasize the individualized care of chronically ill clients as the primary task of primary health care nurses. They refer comparably more often to involving clients in clinical decision-making. In comparison to the other two countries examined here, this indicates a stronger emphasis on clinical tasks in nursing in Spain. This is also confirmed by other research: In primary care in Spain, for example, Advanced Practice Nursing is beginning to establish itself [29, 60, 61], whereas it is largely absent in Brazil and Germany [3, 41]. In Spain a connection with lifeworld-oriented approaches of nursing care [20] is reflected in the model of primary care that foresees nurses’ (as well as general practitioners) long-term support of patients and their families. Consistent with the pattern identified in our study of ‘supporting the integration of illness in clients’ everyday lives’, primary health care nurses in Spain seek to offer support for families throughout the different phases of life with a chronic illness.

### Limitations and further research requirements

This study focused on the practice knowledge and subjective perception of nurses and key informants working in primary care in order to analyze how the promotion of chronically ill patients’ participation and self-management is understood and, in turn, shaped as a task of nursing care. We did not interview patients for our study. Existing studies show, however, that health professionals and patients may, indeed, have different views and expectations of what participation in care can look like and how intensive it should be [11, 62]. Future studies should implement a comparative analysis of nurses’ and clients’ perceptions in order to draw conclusions about the preconditions of ‘successful’ client participation.

Limitations also arise from a methodological point of view: The differences/variations in the way nurses understand and design participation in primary care that emerged in the comparison of the three countries considered here are factors that should be examined in further studies. Moreover, the nurses interviewed in Germany for this study work in primary health care pilot projects, their statements are therefore only transferrable to a very limited extent to the situation of nurses working in, for example, ambulatory nursing services in Germany.

Finally, a further limitation of the study is that our findings allow only few conclusions about how nurses account for the diversity of chronically ill persons; that is, the strategies they actually employ to encourage specific subgroups of chronically ill clients to participate and self-manage their care. If and how clients with chronic illness can and wish to actively participate is, for example, influenced by the progress of their disease. Especially in the late phases of chronic illness, patients not uncommonly expect that nurses make health care-related decisions for them and stand by their side with comfort and advice [63, 64]. Apart from the progress of the disease, factors such as age or sociocultural background also influence the extent to which patients are confident enough to cooperate in the management of their care [65].

## Conclusions

This study provides important findings about nurses’ understanding and shaping of client participation. The results can be used to inform the strategic development of person-centered care models, insofar as they demonstrate how nurses strengthen client participation and self-management in primary care. At the same time, our findings hold the following implications for nursing care practice:

- To enable nurses in primary care to strengthen patients with chronic illness and complex care needs in their participation and self-management, they must have the opportunity to familiarize themselves with the client’s lifeworld, habits, and social environment. This will make it easier for nurses to understand clients’ behavior and health-related decisions, enabling them to negotiate with clients about the implementation of care recommendations.
- Nurses should be sensitive towards ethical challenges that may arise when they want to encourage clients to participate by giving clients greater decision-making and practice autonomy. In this context, they are required to deal with their own feelings of helplessness and insecurity. To counteract moral distress, nurses must also learn to care for themselves. In this respect, they are advised to discuss ethical dilemmas in their teams. Regular structures for discussions – both with clients and other professionals involved in patient care – should become an inherent part of person-centered care models in primary care.

## Data Availability

Given the potentially disclosive nature of entire interview transcripts they will not be made freely publicly available. They will be deposited at Bielefeld University and reasonable requests for secure research access will be considered. Please contact: kerstin.haemel@uni-bielefeld.de.

## Footnotes

1 The standard level of qualification for nurses in Spain and Brazil is the bachelor's degree, however, in Germany specialist vocational training in nursing is the dominant qualification as opposed to university education.

2 The patterns identified in this study apply to the analysis of interviews from all three countries and were discussed with all members of the authorial team.

3 When we use the term "interview partners", this statement includes both practicing nurses and key informants. In places where we explicitly refer to statements by either practicing nurses or key informants, we specify this accordingly.
